# A Novel MICB-Targeting CAR-NK Cells for the Treatment of Pancreatic Cancer

**DOI:** 10.3390/ijms27010500

**Published:** 2026-01-03

**Authors:** Weiyang Jin, Mengying Wang, Jingwei Wang, Jinyi Fan, Jie Fang, Guanghua Yang

**Affiliations:** 1 International Research Center for Biological Sciences, Ministry of Science and Technology, Shanghai Ocean University, Shanghai 201306, China; jin8702181211@163.com (W.J.); wmy5748@163.com (M.W.); tianjiukf@163.com (J.F.); 2 Aquatic Animal Genetics and Breeding Center, Shanghai Ocean University, Shanghai 201306, China; 3 National Aquatic Animal Pathogen Collection Center, Shanghai Ocean University, Shanghai 201306, China; 4 Medical Research Center, Academy of Chinese Medical Sciences, Zhejiang Chinese Medical University, Hangzhou 310050, China; wjw748@163.com; 5 Zhejiang Provincial Laboratory of Experimental Animal’s & Nonclinical Laboratory Studies, Hangzhou Medical College, Hangzhou 310013, China; fangjie3318@163.com

**Keywords:** MICB, IL-15, immunotherapy, CAR-NK, PANC-1

## Abstract

MICB-targeting CAR-NK (chimeric antigen receptor-modified natural killer cells) therapy may serve as off-the-shelf immunotherapy. We designed soluble Anti-MICB-scFv blocks tumor immune evasion targeting the MICB antigen, thereby enhancing CAR-NK cytotoxicity while reactivating endogenous immune attacks against malignancies. The Anti-MICB-CAR includes two Anti-MICB-scFv connected by an F2A linker, the CD8 hinge and transmembrane domain, the 4-1BB co-stimulatory domain, the CD3ζ activation domain, and IL-15. The expression efficiency of Anti-MICB-CAR in NK cells was investigated by flow cytometry; ELISA demonstrated that Anti-MICB-CAR-NK secreted free Anti-MICB-scFv and detected IL-15 secretion. Flow cytometry and CCK8 were utilized to study Anti-MICB-CAR-NK on tumor cell viability. The PANC-1 xenograft model was established in order to elucidate the anti-tumor effects of Anti-MICB-CAR-NK in vivo. In vitro investigations have demonstrated that the treatment of tumor cells with Anti-MICB-CAR-NK supernatant + NK cells or Anti-MICB-CAR-NK cells not only significantly increased the cytotoxic activity of tumor cells, but also secreted and produced higher levels of IL-15, IFN-γ, TNF-α, perforin, and granzyme B compared with NK cells. Anti-MICB-CAR-NK cells exhibit strong cytotoxic activity against tumor cells with high MICB expression. In vivo, Anti-MICB-CAR-NK cells exhibited a substantial inhibitory effect on tumor growth. The IHC results reveal that Anti-MICB-CAR-NK cells show a more pronounced ability to infiltrate the tumor. We demonstrated the successful expression of Anti-MICB-CAR in NK cells, which enhances the anti-tumor activity of NK cells both in vitro and in vivo. This stress ligand-targeting approach provides a promising strategy for solid tumors.

## 1. Introduction

In recent years, cellular immunotherapy has made significant strides in the field of oncology treatment, with the successful application of chimeric antigen receptor T cell (CAR-T) therapy in oncology being particularly notable. By genetically engineering T cells to target specific tumor antigens, CAR-T therapy has demonstrated significant efficacy in relapsed/refractory B-cell and lymphoma [1,2,3]. However, its efficacy in the treatment of solid tumors has fallen far short of expectations, mainly due to the immunosuppressive properties of the tumor microenvironment (TME), tumor antigen heterogeneity, and insufficient CAR-T cell infiltration [4,5,6]. These limitations have prompted researchers to explore more promising alternative cell therapies, among which natural killer cell (NK cell)-based CAR-NK has gradually become a research hotspot [7,8,9].

Compared with CAR-T cells, CAR-NK cells have unique advantages: NK cells are capable of recognizing and eliminating tumor cells without the need for human leukocyte antigen (HLA), thereby reducing the risk of graft-versus-host disease (GVHD) [10,11]. Furthermore, NK cells possess the capacity to engage multiple killing mechanisms through natural receptors (e.g., NKG2D), which may facilitate the overcoming of antigenic escape in solid tumors [12,13,14,15]. However, the development of CAR-NK therapy still faces many challenges, including difficulties in NK cell expansion in vitro, short survival time in vivo, and insufficient persistence of anti-tumor activity [16,17,18]. Solving these problems urgently requires multi-dimensional optimization in terms of target selection, cell engineering modification, and function enhancement strategies [19,20].

In recent years, solid tumor target screening has emerged as a novel research direction [21,22]. Claudin 18.2 is regarded as a viable target for immunotherapy in the treatment of solid tumors, including gastric cancer and pancreatic cancer. The clinical research results confirm that it shows good safety and promising anti-tumor activity in patients with refractory CLDN18.2 positive gastrointestinal tumors [23,24]. Mesothelin is expressed at high levels in a variety of tumors, including malignant pleural mesothelioma, pancreatic cancer, ovarian cancer, and lung cancer. Conversely, its expression is low on the surface of normal pleura, peritoneum, and pericardium. Consequently, mesothelin is regarded as a promising target for cell therapy in the management of solid tumors. Lv, Jiang et al. demonstrated that the infusion of MSLN-CAR-T cells exhibited robust anti-tumor activity, thereby substantiating their potential as a therapeutic modality for GC [25]. EGFRvIII, a glioblastoma-associated antigen, has been identified as a target for immunotherapy. Gene amplification and/or mutations have been observed in approximately 50% of adult primary glioblastoma cases, rendering it an attractive candidate gene for targeted therapy. A clinical trial conducted at the University of Pennsylvania utilized anti-EGFRvIII CAR T cells to treat a cohort of 10 patients diagnosed with glioblastoma multiforme (GBM) that expressed the EGFRvIII protein. The study found this treatment to be well-tolerated and free from evidence of extratumoral toxicity, CRS, or cross-reactivity to wild-type EGFR [26]. Human epidermal growth factor receptor 2 (HER2) is overexpressed in many cancers and chimeric antigen receptor-T (CAR-T) cells targeting HER2 have been shown in clinical trials to be feasible and safe for treating recurrent or refractory central nervous system (CNS) tumors, potentially bringing new therapies for solid tumors [27]. Ganglioside 2 (GD2) is a surface glycolipid antigen that is highly expressed in neuroblastoma, astrocytoma, retinoblastoma, sarcoma, and melanoma, among other cancers. However, its expression is limited in normal tissues and primarily expressed at low levels on neuronal cell bodies and mesenchymal stem cells, rendering it an ideal target for CAR-T [28]. Sirtuins, a group of NAD+- dependent enzymes, have recently been identified as a key factor in the progression and resistance of pancreatic ductal adenocarcinoma (PDAC) and have the potential to become therapeutic targets. Among these, SIRT1 and SIRT2 have attracted the most research attention. The targeting of SIRT1 has been shown to have the potential to overcome drug resistance in invasive malignant tumors and improve treatment outcomes [29]. SIRT2 has been shown to inhibit mismatch repair (MMR) and promote immune escape in colorectal cancer by deacetylating MLH1. Moreover, high expression of SIRT2 is associated with reduced CD8+ T cell infiltration. Consequently, the targeting of SIRT2 has the potential to enhance immune cell infiltration into tumors [30]. In addition, sirtuins have been identified as biomarkers for prostate cancer (PCa) and have the potential to become therapeutic targets [31]. Sirtuins have been shown to play a unique role in the inhibition or promotion of tumor development. The prospect of targeting sirtuins in the context of cellular immunotherapy merits further investigation as a potential avenue for future research.

MICB (MHC class I chain-associated protein B), a stress-induced tumor-associated antigen, consists of α1, α2 (extracellular ligand-binding domain), and α3 (transmembrane/intracellular structural domain). MICB is highly expressed in a variety of solid tumors with restricted expression in normal tissues. It has been demonstrated that MICB binds to the NKG2D receptor of the α1-2 structural domain [32,33]. Not only does this activate the direct killing function of NK cells, but it also recruits CD8+ T cells, CD4+ T cells, and NK cells to co-infiltrate tumor tissues by modulating chemokine secretion in the tumor microenvironment (TME). In animal models, the MICB vaccine significantly increased the enrichment of CD8+ T cells (17.9-fold) and NK cells (38.9-fold) within the tumor, creating a synergistic attack by immune cells. This synergistic effect is particularly groundbreaking for drug-resistant tumors (e.g., MHC-I antigen-presentation-deficient tumors), where MICB, a highly conserved stress molecule, is widely expressed in different tumor types, giving cellular therapeutic oncology drugs targeting MICB antigens the potential to be “generalized”, bypassing the limitations of individualized antigens. Vaccines targeting the MICB antigen have shown significant inhibition of metastatic and drug-resistant tumors in both mouse and rhesus monkey models, with no significant side effects observed [34]. Kerry A Whalen et al. show that the MICA/B monoclonal antibody inhibited the shedding of MICA/B to promote innate immune cell-mediated anti-tumor activity [35]. Approved by the FDA, the antibody-(CLN-619) is currently undergoing Phase I clinical research. Mathieu Bléry et al. show that optimal tumor control was achieved with the MICA/B-ADC format in several solid tumor models, indicating that MICA and MICB are promising targets for cytotoxic immunotherapy [36].

**Figure 1:**
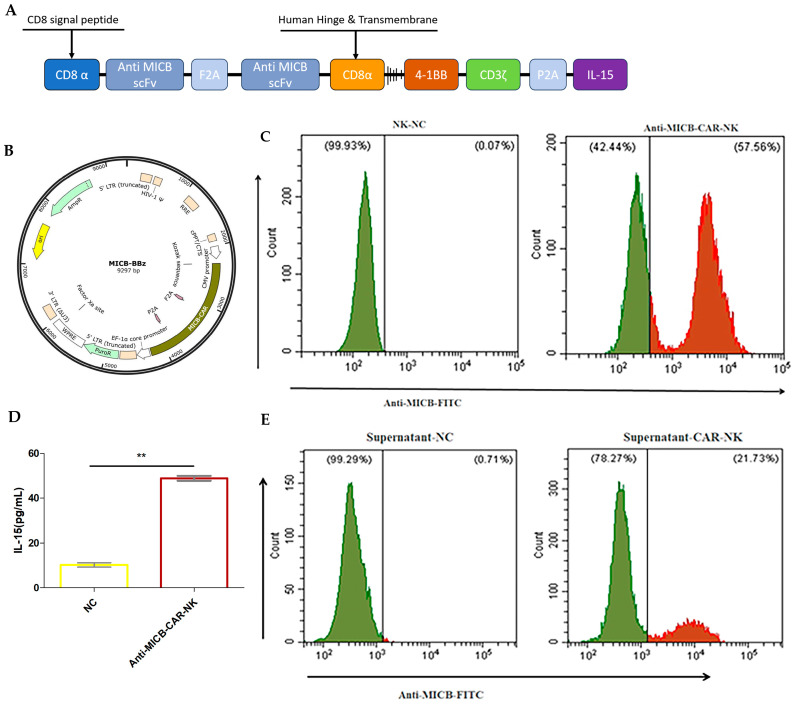
Rational design and construction of the Anti-MICB-CAR. (**A**) Schematic of the Anti-MICB chimeric antigen receptor (Anti-MICB-CAR). (**B**) The plasmid structure of the Anti-MICB chimeric antigen receptor lentiviral vector. (**C**) The expression of Anti-MICB-CAR in NK cells was analyzed via flow cytometry. (**D**) The secretion of IL-15 by Anti-MICB-CAR-NK cells was detected by ELISA. NC: supernatant of 1 × 10^6^ NK cells, Anti-MICB–CAR–NK: supernatant of 1 × 10^6^ Anti-MICB-CAR-NK cells. Supernatants of Anti-MICB-CAR-NK cells were significantly elevated up to 48.88 pg/mL compared to the control NK cell supernatants. *p* = 0.0024. (**E**) The supernatant of 1 × 10^6^ Anti-MICB-CAR-NK cells was filtered and incubated with PANC-1 for 1 h. MICB Protein hFc and IgG1 Fc FITC antibody staining were performed and free Anti-MICB-scFv was detected by flow cytometry. Statistical analysis was performed using a two-tailed Student’s *t*-test; ** *p* < 0.01.

Reducing the expression of MICB on the surface of tumor cells by hydrolyzing the α3 domain of MICB is one of the important mechanisms by which tumor cells conduct immune escape [37,38]. This mechanism provides a direction for the development of novel targeted drugs. Meanwhile, cytokine engineering provides a new idea to enhance the function of CAR-NK [39,40]. However, it will be difficult to activate other immune cells such as T cells, NK cells, and gamma delta T cells in the patient’s body solely with CAR-NK targeting MICB. Therefore, it is imperative to utilize MICB tumor targets to stimulate the activation of additional immune cells within the body, thereby initiating an attack on the tumor cells. Co-expression of IL-15 with CAR structure significantly promotes the survival, proliferation, and anti-tumor activity of NK cells and IL-15 not only maintains the persistence of CAR-NK through autocrine, but also remodels the tumor microenvironment and enhances immune cell infiltration and synergistic killing [41,42,43]. Based on this, we integrated an Anti-MICB-CAR-NK design that targets MICB and secretes IL-15 synergistically (Figure 1A). Firstly, Anti-MICB-scFv targets the MICB α3 domain to prevent MICB shedding, preventing tumor cells from immune escape and prompting the killing of tumor cells by MICB-CAR-NK. Secondly, the designed CAR structure can release free Anti-MICB-scFv, which can bind to MICB on the surface of tumor cells: this will newly reactivate NK and other relevant immune cells in vivo; modulate the secretion of chemokines in the tumor microenvironment (TME); and recruit CD8+ T cells, CD4+ T cells, and NK cells to co-infiltrate tumor tissues. Finally, CAR-NK expression of IL-15 significantly enhances CAR-NK expansion and therapeutic durability. We combine this unique stress ligand-targeting approach with NK cells and take advantage of the extensive high expression of MICB in solid tumors to design a targeted anti-tumor CAR-NK drug that will provide more anti-tumor properties than conventional NK cells, including broad solid tumor responsiveness.

## 2. Results

### 2.1. Rational Design and Construction of the Anti-MICB-CAR

The specific reaction results of the screened Anti-MICBα3 monoclonal antibody are shown in Figures S1 and S2. Anti-MICB monoclonal hybridoma cell line supernatant was mixed with hMICB-293T cells expressing hMICB-293T. The results of the flow assay suggest that the Anti-MICB monoclonal hybridoma cell line exhibited a highly specific response, with a positive rate of 46.0%. Following the purification of the Anti-MICB monoclonal, the Anti-MICB monoclonal antibody’s affinity was assessed for flow cytometry. The results obtained show that the EC50 of the Anti-MICBα3 monoclonal antibody was 0.51 nM, shown in Figure S3.

The Anti-MICB-scFv sequences were obtained via sequencing. The structures of CAR molecules are shown in Figure 1A. The extracellular domain structure consists of two Anti-MICB-scFv linked by F2A and Anti-MICB-scFv binds to the CD8 hinge and transmembrane structural domains, 4-1BB/CD137 co-stimulatory structural domains, and CD3ζ activation structural domains, with IL-15 bound in frame. A CD8α-derived lead peptide was included to promote CAR cell surface expression. The construct was cloned into the lentiviral vector Anti-MICB-scFv-F2A-Anti MIC-scFv-(G4S)3-CD28 TM-4-1BB-CD3ζ-P2A-IL15 (Figure 1B).

We infected constructed lentiviruses into NK cells (Figure S4), as depicted in Figure 1C; flow cytometry results verified the expression of Anti-MICB-CAR in NK cells and the expression rate of Anti-MICB-CAR reached 57.56%. ELISA was performed on the supernatants of Anti-MICB-CAR-NK cells to detect IL-15 in the supernatants, and the IL-15 in the supernatants of Anti-MICB-CAR-NK cells was significantly elevated up to 48.88 pg/mL compared to the control NK cell supernatants (Figure 1D). The supernatants of the Anti-MICB-CAR-NK cells were 0.45 μm filtered and incubated with PANC-1 cells for 1 h. The supernatants were to contain free MICB-scFv flow cytometry and demonstrate the MICB-scFv positivity rate of PANC-1 cells incubated with Anti-MICB-CAR-NK cell supernatants (Figure 1E). The above research confirms the successful production of Anti-MICB-CAR-engineered-NK cells.

### 2.2. Anti-MICB-CAR-NK Cells Demonstrate Potent in Vitro Activity Against Tumors with High Expression of MICB

**Figure 2:**
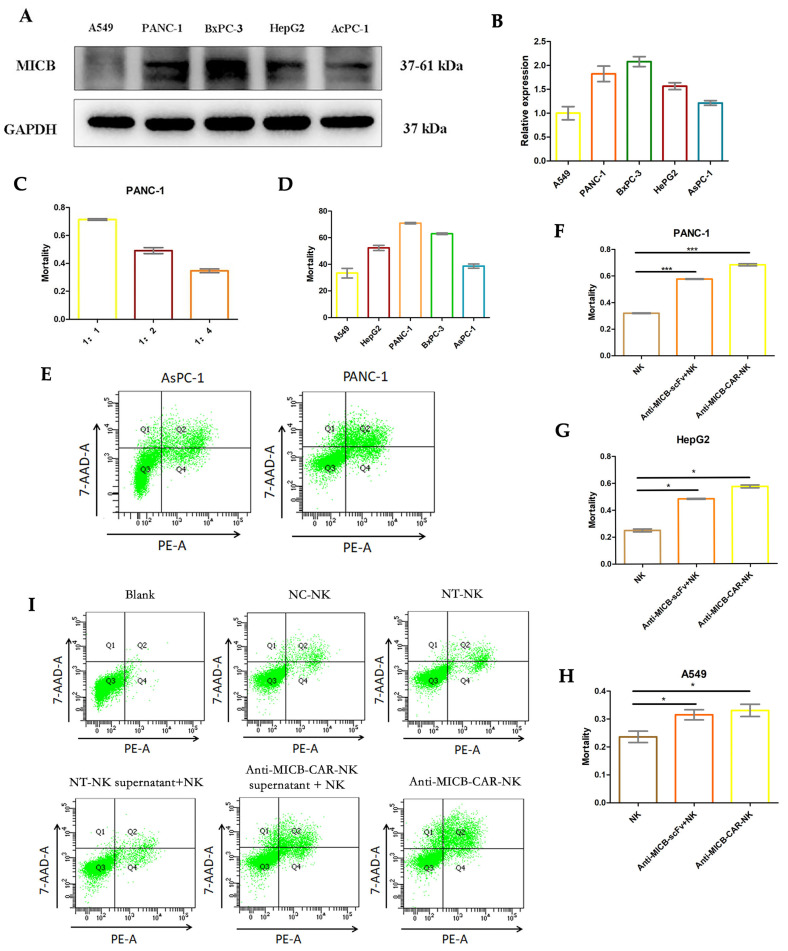
Anti-MICB-CAR-NK cells demonstrate potent in vitro activity against tumors with high expression of MICB. (**A**) Western blot detection of MICB expression in PANC-1, A549, HepG2, BxPC-3, and AsPC-1 human tumor cell lines. (**B**) The qPCR results showed in the MICB gene expression compared to the five tumor cell lines. (**C**) The Effect-to-Target Ratio (E/T Ratio) of Anti-MICB-CAR-NK cells on tumor cell PANC-1. (**D**) The mortality rate of five types of tumor cells treated with Anti-MICB-CAR-NK cells after 24 h with CCK-8 Assay, PANC-1(71.37%), A549(31.78%), HepG2(53.83%), BxPC-3(62.26%), and AsPC-1(39.86%), E/T Ratio 1:1. (**E**) Anti-MICB-CAR-NK treatment of AsPC-1 and PANC–1 tumor cells with differential MICB expression for 24 h by flow cytometry, E/T Ratio 1:1. The results showed that the viability of AsPC-1 was 68.9% and the viability of PANC-1 was 54.6%. (**F**–**H**) PANC-1, HepG2, and A549 tumor cells treated with NK cells, Anti-MICB-CAR-NK cells, and Anti-MICB-CAR-NK supernatant + NK cells for 24 h, E/T Ratio 1:1; CCK8 detects mortality rate. Anti-MICB-CAR-NK cells significantly enhanced the anti-tumor ability compared to NK cells. PANC-1: 68.37%, *p* = 0.0005. HepG2: 57.3%, *p* = 0.0027. A549: 33.18%, *p* = 0.0342. Supernatant of Anti-MICB-CAR-NK cells co-treated tumor cells with NK cells also promoted tumor cell killing compared to NK. PANC-1: 57.66%, *p* = 0.0006. HepG2: 48.21%, *p* = 0.0037. A549: 31.55%, *p* = 0.0446. (**I**) NC-NK cells, non-transduced NK(NT-NK), NT-NK supernatant + NK cells, Anti-MICB-CAR-NK supernatant + NK cells, Anti-MICB-CAR-NK cells treated with PANC-1 tumor cells for 24 h, E/T Ratio 1:1. The viability of each group was detected by flow cytometry and the results showed that NK cells: 89.4%, non-transduced NK(NT-NK): 86.6%, NT-NK supernatant + NK cells: 88.7%, Anti-MICB-CAR-NK supernatant + NK cells: 62.6%, and Anti-MICB-CAR-NK cells: 55.5%. Statistical analysis was performed using a two-tailed Student’s *t*-test; * *p* < 0.05, *** *p* < 0.001.

To evaluate the anti tumor activity of Anti-MICB-CAR-NK in vitro. Western blotting and qPCR was performed to detect the differences in MICB expression in PANC-1, A549, HepG2, BxPC-3, and AsPC-1 (Figure 2A,B). The results show that MICB expression in pancreatic cancer cell lines PANC-1 and BxPC-3 cells was significantly higher than that in lung cancer cell line A549, hepatocellular carcinoma cell line HepG2, and pancreatic cancer cell line AsPC-1, whereas lung cancer cell line A549 had the lowest MICB protein expression. Anti-MICB-CAR-NK and PANC-1 tumor cells were treated with 1:1, 1:2, 1:4 potency-to-target ratio cells for 24 h. The results show that the lethality of Anti-MICB-CAR-NK against PANC-1 was 71.37%, 49.11%, and 34.66%, respectively (Figure 2C). We investigated the killing ability of Anti-MICB-CAR-NK on five tumor cells with differential MICB expression, PANC-1, A549, HepG2, BxPC-3, and AsPC-1. The results show that Anti-MICB-CAR-NK had the strongest killing ability on PANC-1 and BxPC-3 cells with the highest MICB expression, the smallest inhibition on A549 cells with the MICB expression, and the growth inhibition rate of tumor cells Anti-MICB-CAR-NK with significant MICB expression dependence (Figure 2D). Flow cytometry analysis Anti-MICB-CAR-NK treatment significantly enhanced the apoptotic capacity in PANC-1 tumor cells with high MICB expression compared to AsPC-1 cells with low MICB expression (Figure 2E).

Based on the above experimental results, CCK-8 was used to test PANC-1, HepG2, and A549 tumor cells treated with NK cells, Anti-MICB-CAR-NK cells, and Anti-MICB-CAR-NK supernatant + NK cells for 24 h. As shown in Figure 2F–H, it was confirmed that Anti-MICB-CAR-NK cells significantly enhanced the anti-tumor ability compared to NK cells. Anti-MICB-scFv and IL-15 in the supernatant of Anti-MICB-CAR-NK cells co-treated tumor cells with NK cells also promoted tumor cell killing compared to NK.

In order to further research how Anti-MICB-CAR-NK promotes tumor cell apoptosis, treat PANC-1 tumor cells for 24 h with NC-NK cells, non-transduced NK (NT-NK), NT-NK supernatant + NK cells, Anti-MICB-CAR-NK supernatant + NK cells, and Anti-MICB-CAR-NK cells (Figure 2I and Figure S5). The experimental results demonstrated that the viability of PANC-1 tumor cells was ‌55.55%‌ after treatment with ‌Anti-MICB-CAR-NK cells‌ and the co-culture of ‌Anti-MICB-CAR-NK supernatant + NK cells‌ also significantly reduced the viability of PANC-1 cells. In contrast, when treated with ‌NC-NK cells‌, ‌non-transduced NK (NT-NK)‌, or ‌NT-NK supernatant + NK cells‌, the survival rate of PANC-1 cells remained above ‌80%‌. These findings indicate that ‌Anti-MICB-CAR-NK cells‌ and ‌Anti-MICB-CAR-NK supernatant + NK cells‌ meaningfully suppressed PANC-1 cell survival and promoted apoptosis.

### 2.3. Anti-Tumor Mechanism Induced by Anti-MICB-CAR-NK Cells

**Figure 3:**
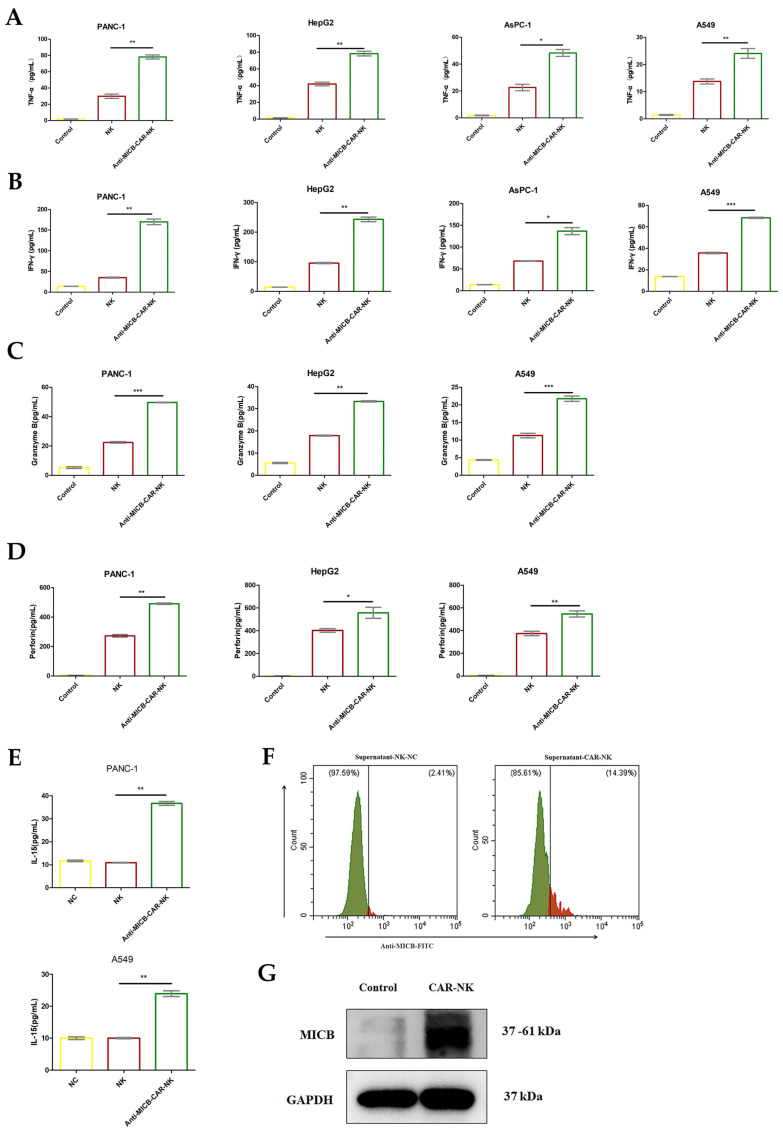
The anti-tumor mechanism induced by Anti-MICB-CAR-NK cells. (**A**–**D**) The release of perforin, granzyme B, TNF-α, and IFN-γ from PANC-1, HepG2, and A549 cells. NK cells and Anti-MICB-CAR-NK cells were treated with tumor cells for 24 h, with an E/T ratio of 1:1. Control represents complete medium cultivation. The supernatant was collected and detected by ELISA. Compared to the NK group, the Anti-MICB-CAR-NK group exhibited significantly higher levels of TNF-α expression (PANC-1, *p* = 0.0072; AsPC-1, *p* = 0.035; HepG2, *p* = 0.0076; A549, *p* = 0.0066) and IFN-γ expression (PANC-1, *p* = 0.0072; AsPC-1, *p* = 0.0162; HepG2, *p* = 0.002; A549, *p* = 0.0006) following treatment with PANC-1, AsPC-1, HepG2, and A549 tumor cells. Similarly, Granzyme B expression levels were significantly elevated in the Anti-MICB-CAR-NK group (PANC-1, *p* = 0.0007; HepG2, *p* = 0.0023; A549, *p* = 0.0004), as were Perforin expression levels (PANC-1, *p* = 0.0021; HepG2, *p* = 0.042; A549, *p* = 0.0022) after treatment with PANC-1, HepG2, and A549 tumor cells. Additionally, the Anti-MICB-CAR-NK group demonstrated significantly increased IL-15 expression following treatment with PANC-1 and A549 tumor cells (PANC-1, *p* = 0.0012; A549, *p* = 0.0025) compared to the NK group. (**E**,**F**) Elisa and flow cytometry were used to detect the levels of IL-15 and Anti-MICB-scFv in the tumor cells’ supernatants treated with Anti-MICB-CAR-NK cells for 24 h. (**G**) Study on MICB expression in PANC-1 treated with Anti-MICB-CAR-NK cells for 24 h using Western blot technique. Statistical analysis was performed using a two-tailed Student’s *t*-test; * *p* < 0.05, ** *p* < 0.01, *** *p* < 0.001.

The release of perforin, granzyme B, TNF-α, and IFN-γ from PANC-1, HepG2, and A549 cells was measured using ELISA (Figure 3A–D). The levels of cytokines released by Anti-MICB-CAR-NK cells post-killing PANC-1, HepG2, and A549 cells were significantly higher than those released by NK cells.

Figure 3E,F show that after the treatment of NK and Anti-MICB-CAR-NK cell, the content of IL-15 were detected using ELISA and flow cytometry and the results show that, compared with the NK group, the Anti-MICB-CAR-NK cell group secreted a large amount of Anti-MICB-scFv and IL-15. Anti-MICB-CAR-NK cells activate the tumor-killing ability of NK cells by targeting MICB and preventing MICB shedding; IL-15 enhances the activity of Anti-MICB-CAR-NK cells. As shown in Figure 3G, Anti-MICB-CAR-NK cells after PANC-1 revealed that MICB expression was significantly elevated, which predicts that Anti-MICB-CAR-NK cells expressing Anti-MICB-scFv blocked the shedding of MICB protein from the tumor cells and increased the killing ability of NK cells on tumor.

### 2.4. Anti-MICB-CAR-NK Cells Exhibit Tumor Regression in the PANC-1 Xenograft Model

**Figure 4:**
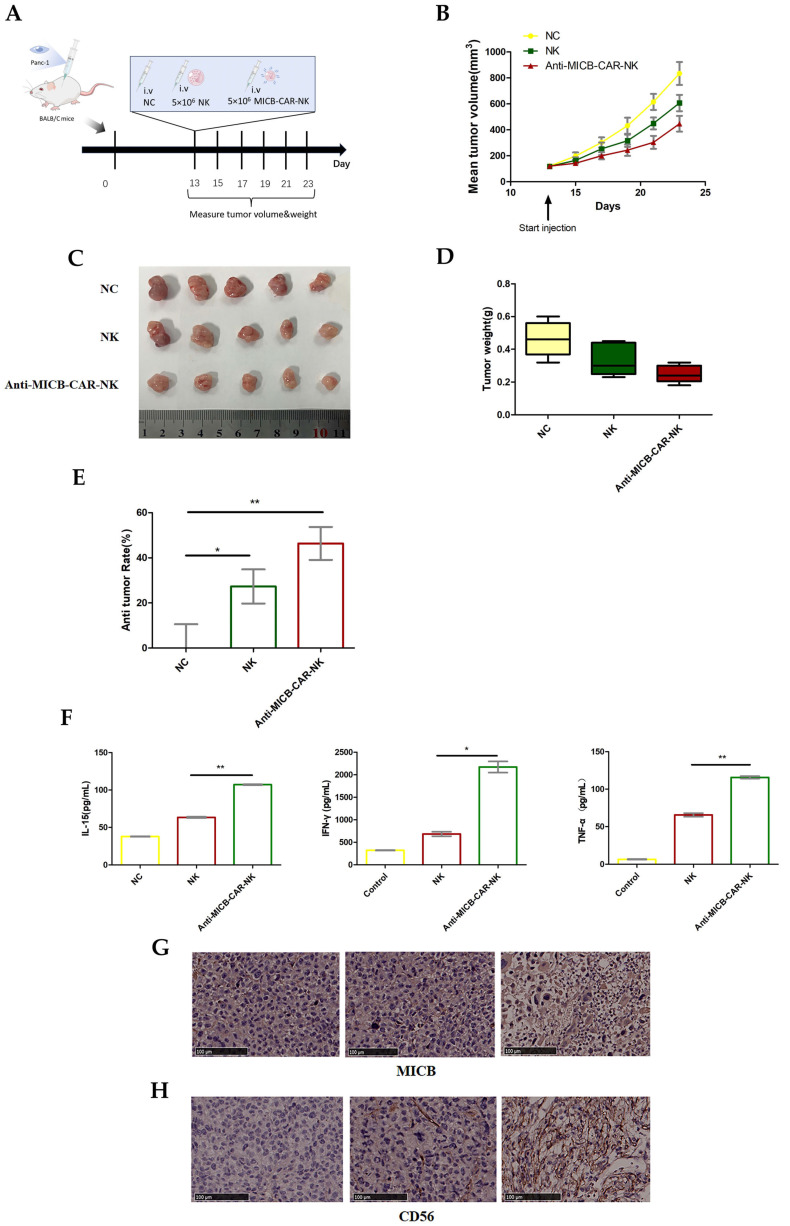
Anti-MICB-CAR-NK cells exhibit tumor regression in the PANC-1 Xenograft Model. (**A**) Flow chart of Anti-MICB-CAR-NK cells treatment for PANC-1 Transplanted Tumors. On the 0th day, PANC-1 cells were inoculated and on the 13th day, PBS, NK cells, and Anti-MICB-CAR-NK cells were injected into mice via tail vein at a dose of 5 × 10^6^ cells per mouse. Record tumor growth observed every other day. (**B**) Growth curve of mouse transplanted tumor. (**C**,**D**) Resected tumors from each group were imaged and Weighed at the end of the experiment. NC: 834.31 ± 197.26 (mg), NK: 606.69 ± 141.37 (mg), Anti-MICB-CAR-NK: 447.32 ± 136.13 (mg). (**E**) Anti-tumor rate of Anti-MICB-CAR-NK cells against PANC-1 transplanted tumors. The tumor inhibition rate of NK compared to NC was 27.28%, *p* = 0.07. The tumor inhibition rate of Anti-MICB-CAR-NK compared to NC was 46.38%, *p* = 0.007. (**F**) Mouse serum was collected at the end of the experiment and the amounts of IL-15, IFN-γ, and TNF-α were measured by ELISA. The IL-15, IFN—γ, and TNF—α in the Anti-MICB-CAR-NK group were significantly increased compared to the NK group, with a *p*-value of 0.0013 for IL-15, 0.013 for IFN—γ, and 0.003 for TNF—α. (**G**,**H**) Mouse tumor tissues were analyzed by immunohistochemistry for MICB expression and NK cell infiltration within the tumor tissue. Statistical analysis was performed using a two-tailed Student’s *t*-test; * *p* < 0.05, ** *p* < 0.01.

To further evaluate the anti-tumor efficacy of Anti-MICB-CAR-NK, the PANC-1 xenograft model was established and treated with PBS, NK cells, and Anti-MICB-CAR-NK cells. As shown in Figure 4A–E, NK and Anti-MICB-CAR-NK significantly decreased tumor growth by 27.28% and 46.38%, respectively (with no obvious effects on body weight). To further confirm the efficacy of Anti-MICB-CAR-NK on the PANC-1 xenograft model, ELISA detection of IL-15 in serum. IHC analysis tumors were collected at the end of the animal studies. The expression of IL-15 in the serum of Anti-MICB-CAR-NK-treated mice was observed to be about two folds higher than that in the NK and PBS groups, respectively (Figure 4F). As illustrated in Figure 4G, the expression of MICB in the tumor tissue of the Anti-MICB-CAR-NK group was significantly higher than that of the NK and NC groups. This result indicates that the Anti-MICB-CAR-NK treatment enhanced the ability of NK cells to bind with tumor cells. Furthermore, increased numbers of NK cells were observed in the IHC-stained tissue from the Anti-MICB-CAR-NK-treated tumors (Figure 4H). All these experimental results on the PANC-1 xenograft model further confirmed the anti-tumor efficacy of Anti-MICB-CAR-NK and the possible anti-tumor mechanism.

## 3. Discussion

Recent studies in the rapidly evolving field of CAR-immune cell therapy have shown that primary CAR-NK cells are safe and effective [44]. To develop NK cell therapy concepts that address malignant solid tumors, there is an urgent need to investigate CAR-NK cells that target these tumors.

In this study, we rationally designed Anti-MICB-CAR, a new strategy for targeted therapy of solid tumors show that our screened monoclonal antibody, with hMICBα3 as antigen has excellent affinity for hMICB. We analyzed the sequence of Anti-MICB α3 monoclonal antibody gene sequencing, rationally designed Anti-MICB-CAR, and successfully transduced Anti-MICB-CAR into NK cells to stably and efficiently express CAR. This novel Anti-MICB-CAR-NK has the following features: First, it has a unique CAR structure that targets the MICBα3 structural domain of the NKG2D ligand. This structure recognizes MICBα3 antigenic epitopes expressed by cancer cells. It prevents MICB detachment and inhibits tumor cells from evading immune monitoring. Second, the CAR structure is designed to release free Anti-MICB-scFv, which binds to MICB on the surface of distal tumor cells. This allows the body’s immune cells, including NK cells, NKT cells, γδ T cells, and CD8+ T cells, to reactivate tumor cell killing through NKG2D binding. Third, Anti-MICB-CAR-NK cells that secrete IL-15 can significantly improve the expansion and persistence of CAR-NK therapy in vivo. Fourth, MICB is highly expressed in various solid tumors, making Anti-MICB CAR NK cells versatile for solid tumor therapy. Badrinath et al. used this target for tumor vaccine development [34].

In vitro, cytotoxicity assays demonstrated that Anti-MICB-CAR-NK cells surpass parental NK cells in eliminating various cancer lines, particularly PANC-1 cells with high MICB expression, and even show improvement in A549 cells with low expression levels. As the expression of MICB protein by tumor cell lines increased, Anti-MICB-CAR-NK cells exhibited a stronger capacity to kill tumor cells, thereby demonstrating a clear MICB expression-dependent growth inhibition of tumor cells Anti-MICB-CAR-NK. Next, we clarify the underlying mechanisms by which Anti-MICB-CAR-NK may confer advantages. This could involve exploring how targeting MICB might overcome immune evasion strategies employed by cancer cells, to more robust NK cell activation. Anti-MICB-scFv, when targeting the MICBα3 domain, has been shown to bind to MICB proteins on the surface of tumor cells with high efficiency. This binding prevents the hydrolytic shedding of these proteins and inhibits the evasion of immune surveillance. Consequently, this results in the killing of tumor cells by natural killer (NK) cells. It was determined that Anti-MICB-CAR-NK cells co-treated with pancreatic cancer tumor cell line PANC-1 for 24 h secreted significant quantities of Anti-MICB-scFv and IL-15. This phenomenon led to an increased binding of tumor cells by secreted Anti-MICB-scFv, subsequently inducing the lysis of tumor cells by immune cells. The secretion of IL-15 was found to enhance the survival and persistence of Anti-MICB-CAR-NK cells and NK cells. The Western blot experiments demonstrated that MICB expression was significantly elevated in Anti-MICB-CAR-NK treated PANC-1. This finding suggests that the binding of Anti-MICB-scFv to MICB on the surface of tumor cells hindered its shedding, leading to substantial augmentation in MICB protein expression in Anti-MICB-CAR-NK cell-treated PANC-1 compared to NK cell-treated PANC-1. Anti-MICB-CAR-NK cells not only increased cancer cell killing but also secreted higher levels of cytokines such as IFN-γ, TNF-α, perforin, and granzyme B. By releasing perforin and granzyme B to trigger apoptosis of tumor cells, while secreting IFN-γ and TNF-α to regulate the immune microenvironment, it forms an efficient anti-tumor mechanism with multiple levels and pathways.

In vivo, Anti-MICB-CAR-NK cells significantly controlled the growth of PANC-1 transplanted tumors and increased cytokine secretion. Blocking the hydrolytic shedding of MICB may have promoted the secretion of perforin and granzyme B, leading to more NK cell infiltration of the tumor, which correlates with the activation of immune anti-tumor by MICB binding to ligand NKG2D. Subsequent validation of these results in animal models will provide indisputable evidence that our designed Anti-MICB-CAR-NK cells can effectively trigger autoimmunity within the anti-tumor mechanism, resulting in the elimination of multiple solid tumors. Due to the limitations of the PANC-1 transplantation tumor model designed for the present study, the anti-tumor effect of the Anti-MICB-CAR-NK cell function was the primary focus, without direct investigation of the immune activation status of Anti-MICB-CAR-NK cells in vivo, as well as the analysis of persistence. The importance of these factors is fully recognized for future studies. The current dataset offers significant insights into the therapeutic potential of Anti-MICB-CAR-NK cells, thereby identifying promising avenues for further research. It is important to note that the administration of overt immunotherapy for solid tumors necessitates a multifaceted strategy that extends beyond the enhancement of cell-killing capacity to encompass the modulation of the tumor microenvironment to promote immune cell infiltration.

The present study indicates that Anti-MICB-CAR has been successfully expressed in primary natural killer (NK) cells and secretes free Anti-MICB-scFv and interleukin-15 (IL-15), demonstrating strong tumor-killing ability. The Anti-MICB-CAR-NK cells hold considerable promise for the treatment of pancreatic cancer with high MICB expression. This assertion is supported by compelling evidence, which is the robust anti-tumor efficacy of Anti-MICB-CAR-NK cells.

## 4. Materials and Methods

### 4.1. Cell Lines and Cell Culture

Human lung cancer cell line A549, human liver cancer cell line HepG2, human pancreatic cancer cell lines AsPC-1, BxPC-3, and PANC-1 and HEK293T cells were procured from the Chinese Academy of Sciences Typical Culture Collection Committee Cell Bank (CCTCC), Shanghai, China. Human primary natural killer (NK) cells were isolated from the blood of healthy donors and human peripheral blood mononuclear cells (PBMCs) were provided by Milestone Biotechnologies, Shanghai, China.

Human tumor cells were cultured at 37 °C and 5% CO_2_. This included A549, HepG2, 293T, AsPC-1, BxPC-3, and PANC-1. Peripheral blood mononuclear cells (hPBMCs) were obtained from the peripheral blood of healthy individuals. AsPC-1, BxPC-3, and PANC-1 cells were cultured in 1640 medium supplemented with 10% fetal bovine serum (FBS). A549, HepG2, PANC-1, and 293T cells were cultured in DMEM medium containing 10% FBS. NK cells were isolated from human peripheral blood mononuclear cells (hPBMC). After incubating hPBMC at 37 °C, NK cells were isolated, cultured, and expanded using the NK cell serum-free culture kit provided by Youkang Biotechnology (catalog number: AN0104), Beijing, China. By day 11 of NK cell culture, the proportion of NK cells (CD3-CD56+) reached over 95%, enabling subsequent research.

### 4.2. Preparation and Sequence Analysis of Anti-MICB α 3 Monoclonal Antibody

DNA-mediated immunity was established in C57 mice with MICB gene knockout. Mice demonstrating substantial immune responses were selected for intraperitoneal challenge immunization. Spleens were harvested 3–4 days post-immunization, ground using a 70 μm sieve, fused with SP2/0 cells via PEG-mediated fusion, and plated. Hybridoma screening was performed using hMICB-expressing CHO cells (provided by Nest Biotechnology (Hangzhou) Co., Ltd., Hangzhou, China) and subcloned to obtain Anti-MICB monoclonal hybridoma cells. The peritoneal cavity of mice was injected with Anti-MICB monoclonal hybridoma cells. The ascites were collected, isolated, and purified, and the Anti-MICB α3 monoclonal antibody was acquired. The variable and constant regions of the Anti-MICB α3 monoclonal antibody were identified via gene sequencing.

### 4.3. CAR Construction, Lentivirus Production

The scFv sequence of the Anti-MICBα3 monoclonal antibody, which functions as an antigen recognition domain, is connected through a G4S linker (GGGGSGGGGGGGGS). Constructed Anti-MICB-CAR, the extracellular domain structure consists of two MICB-scFv sequences connected by F2A. The Anti-MICB-scFv demonstrates binding to the CD8 hinge and transmembrane domains, the 4-1BB co-stimulatory domain, and the CD3 Zeta activation domain, as well as IL-15 within the framework. The CAR contains a lead peptide derived from CD8 α, which is known to promote CAR cell surface expression. The construct was subcloned into the lentiviral vector, which contained the Anti-MICB-scFv-F2A-Anti-MICB-scFv-(G4S)3-CD28TM-4-1BB-CD3Zeta-P2A-IL15.The third-generation self-inactivating lentiviral vector was generated by transiently transfecting HEK293T cells with polyethyleneimine(PEI). The infected cell line obtained transducing natural killer (NK) cells with virus supernation and are henceforth designated Anti-MICB-CAR-NK.

### 4.4. Transduction of NK Cells

On the 11th day of NK cell culture, the proportion of NK cells (CD3-CD56+) reached over 99 and NK cells were transduced with lentiviral particles. The co-infective agent Vectofusin-1 (Miltenyi Biotec; Cat: 130-111-163, Shanghai, China) with lentiviral particles at a final concentration of 5 μg/mL, with a total volume of 100 μL, were incubated at room temperature for 7 min. The mixture to 100 μL of NK cell solution with a concentration of 1 × 10^6^ cells/mL (using Youkang serum-free amplification medium containing IL-12, IL-15, and IL-18) is centrifuged at 400× *g* centrifugal force for 0.5 h, and then incubated in a 37 °C incubator for 1 h. Place the cells into a 48-well plate and culture normally. Culture medium containing 5% blood replacement after 24 h.

Flow cytometry analysis of the content of NK cells isolated from human peripheral blood mononuclear cells (hPBMC). 10^6^ NK cells centrifugation, resuspend in 1 mL PBS, 20 μL of serum for 45 min, 20 μL of anti-human CD56 Antibody APC (Biolegend, Cat: 362503, San Diego, CA, USA), and 20 μL of FITC anti-human CD3 antibody (Biolegend, Cat: 300305, San Diego, CA, USA), and incubate in the dark for 1.5 h.

Anti-MICB-CAR expression on gene-modified NK cells using flow cytometry analyzed with an Anti-MICB-CAR Detection Reagent, containing a recombinantly expressed fusion protein consisting of the human MICB extracellular domains and a specifically mutated human lgG1 Fc region (Sino Biological, Recombinant Human MICB Protein-His & hFc Tag, Cat:10759-H03H, Beijing, China) and secondary addition of anti-Human IgG1 Fc-FITC-antibody (Invitrogen, anti-Human IgG1 Fc Secondary Antibody, Cat: A-10631, Carlsbad, CA, USA).

### 4.5. Western Blot Analysis

For Western blot analysis, cells were washed twice with 1 × PBS, before adding protease inhibitors to the cell lysis buffer to preserve target proteins in the cell extract. Lysates were analyzed under denaturing conditions on 10% SDS-PAGE. The proteins on the SDS-PAGE gel were then transferred onto PVDF membranes. Membranes were blotted with transfer buffer for 45 min at room temperature and blocked with 5% skim milk for 1 h. The membranes were then incubated with an Anti-MICB antibody (abcam, ab300485, 1:1500, Hangzhou, Zhejiang, China) overnight with light shaking at 4 °C, before washing with TBST and incubating with HRP-conjugated anti-rabbit IgG secondary antibody (FUDE BIO, FD0155, 1:5000, Hangzhou, Zhejiang, China) in 5% skim milk for 2 h. After TBST cleaning three times, for 10 min, an imager was used for exposure.

### 4.6. Cell Counting Kit-8

Cells were plated in 96-well plate (5000 cells per well) and treated with the indicated cell concentration of Anti-MICB-CAR-NK for 24 h. Cell viability was assayed CCK8 kit (Dojindo Laboratories, Kumamoto, Japan) according to the manufacturer’s manual. The absorbance at 450 nm was determined via Thermo Varioskan Flash.

### 4.7. ELISA

ELISA of Perforin, Granzyme B, IFN γ, TNF α, and IL-15 were purchased from BBI, Shanghai, China. Standard and serum samples (100 μL) were added to a 96-well plate, 50 μL each enzyme added to each well for 1 h incubation, the liquid removed, the plate washed times, 50 μL substrate added and incubated in the dark for 15 min, and 50 μL termination solution added. The OD value was measured at a wavelength of 450 nm.

### 4.8. Immunohistochemistry Analysis of CD56 and MICB

Tumor specimens were removed from mice, fixed in 4% paraformaldehyde for 24 h, progressively dehydrated, and embedded in paraffin. The slices of tissues were immunostained with MICB, CD56 antibodies. Micrographs were acquired using a C13210-01 NanoZoomer S60, Hamamatsu, Japan.

### 4.9. Xenograft Tumor Models

Animal experiments were in accordance with local guidelines for the care of laboratory animals Animal Experimental Center, Zhejiang Academy of Medical Sciences and were approved by the ethics for research on laboratory animal use of the Zhejiang Academy of Medical Sciences. Six-week-old nude mice were maintained under specific pathogen-free (SPF) conditions. To establish the PANC-1 xenograft model, 5 × 10^6^ PANC-1 cells were resuspended in 100 μL of PBS and inoculated subcutaneously into mice. Upon reaching a tumor volume of 80–100 mm^3^, the tumor-bearing mice were randomized and evenly distributed into three groups (*n* = 5 each): PBS, NK cells, and Anti-MICB-CAR-NK cells. Mice received injections via the tail vein, 1 × 10^6^ NK cells, Anti-MICB-CAR-NK cells, or 100 μL of PBS alone. The tumor volumes were monitored throughout the study. Upon study termination, the mice were euthanized cervical dislocation. Then the tumors were harvested blood samples were collected for cytokine analysis ELISA.

### 4.10. Statistical Analysis

Statistically significant differences between the experimental and untreated control groups were detected using Student’s unpaired t-tests. Each set of the experiment was repeated at least three times. *p* < 0.05 was considered statistically significant. Statistical analysis was performed using GraphPad PRISM software (10.5.0, GraphPad Software, Inc., San Diego, CA, USA).

## 5. Conclusions

In this study, we demonstrate that the expression of a novel Anti-MICB-CAR in natural killer (NK) cells results in significant in vitro cellular toxicity and in vivo tumor inhibition of PANC-1 human pancreatic cancer cells. The Anti-MICB-CAR-NK possesses two key highlights: First, it secretes a free Anti-MICB-scFv fragment, which prevents the shedding of MICB from the tumor cell surface and may thereby activate immune cells such as T cells, γδT cells, and NK cells to attacking the tumor. Second, the NK cells are genetically engineered to specifically recognize MICB-expressing tumor cells, thereby enhancing the cytotoxic activity of the infused Anti-MICB-CAR-NK cells. Additionally, the secretion of IL-15 prolongs the in vivo persistence of Anti-MICB-CAR-NK cells. The rapid clearance of PANC-1 cells may be attributed to the ability of Anti-MICB-CAR-NK cells and free Anti-MICB-scFv to block MICB detachment from the tumor cells, allowing NKG2D to bind to MICB and activate tumor killing in vivo. MICB binding activates tumor killing through the immune cells in vivo. In addition, this approach provides a meaningful theoretical basis for the treatment of solid tumors with Anti-MICB-CAR-NK cells and offers a potential pathway for the clinical treatment of solid tumors such as pancreatic cancer.

## Data Availability

No datasets were generated or during the current study.

## Supplementary Materials

The following supporting information can be downloaded at: https://www.mdpi.com/article/10.3390/ijms27010500/s1.

## References

[1] Strati P. (2022). CAR T-cell therapy: Which product for which patient?. Blood.

[2] Amini L., Silbert S.K., Maude S.L., Nastoupil L.J., Ramos C.A., Brentjens R.J., Sauter C.S., Shah N.N., Abou-El-Enein M. (2022). Preparing for CAR T cell therapy: Patient selection, bridging therapies and lymphodepletion. Nat. Rev. Clin. Oncol..

[3] Qi Y., Zhao M., Hu Y., Wang Y., Li P., Cao J., Shi M., Tan J., Zhang M., Xiao X. (2022). Efficacy and safety of CD19-specific CAR T cell-based therapy in B-cell acute lymphoblastic leukemia patients with CNSL. Blood.

[4] Sterner R.C., Sterner R.M. (2021). CAR-T cell therapy: Current limitations and potential strategies. Blood Cancer J..

[5] Du B., Qin J., Lin B., Zhang J., Li D., Liu M. (2025). CAR-T therapy in solid tumors. Cancer Cell.

[6] Zhao Y., Chen J., Andreatta M., Feng B., Xie Y.-Q., Wenes M., Wang Y., Gao M., Hu X., Romero P. (2024). IL-10-expressing CAR T cells resist dysfunction and mediate durable clearance of solid tumors and metastases. Nat. Biotechnol..

[7] Peng L., Sferruzza G., Yang L., Zhou L., Chen S. (2024). CAR-T and CAR-NK as cellular cancer immunotherapy for solid tumors. Cell. Mol. Immunol..

[8] Wang W., Liu Y., He Z., Li L., Liu S., Jiang M., Zhao B., Deng M., Wang W., Mi X. (2024). Breakthrough of solid tumor treatment: CAR-NK immunotherapy. Cell Death Discov..

[9] Maia A., Tarannum M., Lérias J.R., Piccinelli S., Borrego L.M., Maeurer M., Romee R., Castillo-Martin M. (2024). Building a Better Defense: Expanding and Improving Natural Killer Cells for Adoptive Cell Therapy. Cells.

[10] Lamers-Kok N., Panella D., Georgoudaki A.M., Liu H., Özkazanc D., Kučerová L., Duru A.D., Spanholtz J., Raimo M. (2022). Natural killer cells in clinical development as non-engineered, engineered, and combination therapies. J. Hematol. Oncol..

[11] Liu E., Tong Y., Dotti G., Shaim H., Savoldo B., Mukherjee M., Orange J., Wan X., Lu X., Reynolds A. (2018). Cord blood NK cells engineered to express IL-15 and a CD19-targeted CAR show long-term persistence and potent antitumor activity. Leukemia.

[12] Prager I., Watzl C. (2019). Mechanisms of natural killer cell-mediated cellular cytotoxicity. J. Leukoc. Biol..

[13] Jhunjhunwala S., Hammer C., Delamarre L. (2021). Antigen presentation in cancer: Insights into tumour immunogenicity and immune evasion. Nat. Rev. Cancer.

[14] Mahr A.R., Bennett-Boehm M.M.C., Rothemejer F.H., Weber I.S., Regan A.K., Franzen J.Q., Bisson C.R., Truong A.N., Olesen R., Schleimann M.H. (2024). TLR9 agonism differentially impacts human NK cell-mediated direct killing and antibody-dependent cell-mediated cytotoxicity. Sci. Rep..

[15] Wang D., Dou L., Sui L., Xue Y., Xu S. (2024). Natural killer cells in cancer immunotherapy. MedComm.

[16] Xie G., Dong H., Liang Y., Ham J.D., Rizwan R., Chen J. (2020). CAR-NK cells: A promising cellular immunotherapy for cancer. EBioMedicine.

[17] Li T., Niu M., Zhang W., Qin S., Zhou J., Yi M. (2024). CAR-NK cells for cancer immunotherapy: Recent advances and future directions. Front. Immunol..

[18] Zhang L., Meng Y., Feng X., Han Z. (2022). CAR-NK cells for cancer immunotherapy: From bench to bedside. Biomark. Res..

[19] Gong Y., Klein Wolterink R.G.J., Wang J., Bos G.M.J., Germeraad W.T.V. (2021). Chimeric antigen receptor natural killer (CAR-NK) cell design and engineering for cancer therapy. J. Hematol. Oncol..

[20] Khawar M.B., Sun H. (2021). CAR-NK Cells: From Natural Basis to Design for Kill. Front. Immunol..

[21] Chong C., Coukos G., Bassani-Sternberg M. (2022). Identification of tumor antigens with immunopeptidomics. Nat. Biotechnol..

[22] Moravec Z., Zhao Y., Voogd R., Cook D.R., Kinrot S., Capra B., Yang H., Raud B., Ou J., Xuan J. (2025). Discovery of tumor-reactive T cell receptors by massively parallel library synthesis and screening. Nat. Biotechnol..

[23] Qi C., Xie T., Zhou J., Wang X., Gong J., Zhang X., Li J., Yuan J., Liu C., Shen L. (2023). CT041 CAR T cell therapy for Claudin18.2-positive metastatic pancreatic cancer. J. Hematol. Oncol..

[24] Qi C., Gong J., Li J., Liu D., Qin Y., Ge S., Zhang M., Peng Z., Zhou J., Cao Y. (2022). Claudin18.2-specific CAR T cells in gastrointestinal cancers: Phase 1 trial interim results. Nat. Med..

[25] Lv J., Zhao R., Wu D., Zheng D., Wu Z., Shi J., Wei X., Wu Q., Long Y., Lin S. (2019). Mesothelin is a target of chimeric antigen receptor T cells for treating gastric cancer. J. Hematol. Oncol..

[26] Thokala R., Binder Z.A., Yin Y., Zhang L., Zhang J.V., Zhang D.Y., Milone M.C., Ming G.-L., Song H., O’rourke D.M. (2021). High-Affinity Chimeric Antigen Receptor With Cross-Reactive scFv to Clinically Relevant EGFR Oncogenic Isoforms. Front. Oncol..

[27] Vitanza N.A., Johnson A.J., Wilson A.L., Brown C., Yokoyama J.K., Künkele A., Chang C.A., Rawlings-Rhea S., Huang W., Seidel K. (2021). Locoregional infusion of HER2-specific CAR T cells in children and young adults with recurrent or refractory CNS tumors: An interim analysis. Nat. Med..

[28] Ciccone R., Quintarelli C., Camera A., Pezzella M., Caruso S., Manni S., Ottaviani A., Guercio M., Del Bufalo F., Quadraccia M.C. (2024). GD2-Targeting CAR T-cell Therapy for Patients with GD2+ Medulloblastoma. Clin. Cancer Res..

[29] Chouhan S., Kumar A., Muhammad N., Usmani D., Khan T.H. (2024). Sirtuins as Key Regulators in Pancreatic Cancer: Insights into Signaling Mechanisms and Therapeutic Implications. Cancers.

[30] Yi F., Zhang Y., Wang Z., Wang Z., Li Z., Zhou T., Xu H., Liu J., Jiang B., Li X. (2021). The deacetylation-phosphorylation regulation of SIRT2-SMC1A axis as a mechanism of antimitotic catastrophe in early tumorigenesis. Sci. Adv..

[31] Chouhan S., Muhammad N., Usmani D., Khan T.H., Kumar A. (2024). Molecular Sentinels: Unveiling the Role of Sirtuins in Prostate Cancer Progression. Int. J. Mol. Sci..

[32] Petersdorf E.W., Shuler K.B., Longton G.M., Spies T., Hansen J.A. (1999). Population study of allelic diversity in the human MHC class I-related MIC-A gene. Immunogenetics.

[33] Fuertes M.B., Domaica C.I., Zwirner N.W. (2021). Leveraging NKG2D Ligands in Immuno-Oncology. Front Immunol..

[34] Badrinath S., Dellacherie M.O., Li A., Zheng S., Zhang X., Sobral M., Pyrdol J.W., Smith K.L., Lu Y., Haag S. (2022). A vaccine targeting resistant tumours by dual T cell plus NK cell attack. Nature.

[35] Whalen K.A., Henry C.C., Mehta N.K., Rakhra K., Yalcin S., Meetze K., Gibson N.W., Baeuerle P.A., Michaelson J.S. (2025). CLN-619, a MICA/B monoclonal antibody that promotes innate immune cell-mediated antitumor activity. J. Immunother. Cancer.

[36] Bléry M., Mrabet-Kraiem M., Morel A., Lhospice F., Bregeon D., Bonnafous C., Gauthier L., Rossi B., Remark R., Cornen S. (2021). Targeting MICA/B with cytotoxic therapeutic antibodies leads to tumor control. Open Res. Eur..

[37] Wang R., Wu J., Lin Y., Xiao Y., Yang B., Yao S., Pan T., Fu Z., Li S., Wang C. (2025). An epitope-directed mRNA vaccine inhibits tumor metastasis through the blockade of MICA/B α1/2 shedding. Cell Rep. Med..

[38] Goulding J., Yeh W.I., Hancock B., Blum R., Xu T., Yang B.-H., Chang C.-W., Groff B., Avramis E., Pribadi M. (2023). A chimeric antigen receptor uniquely recognizing MICA/B stress proteins provides an effective approach to target solid tumors. Med.

[39] Guo C., Dong M., Wang X., Yu J., Jin X., Cheng S., Cui F., Qian Y., Bao Q., Zhi L. (2024). A novel MICA/B-targeted chimeric antigen receptor augments the cytotoxicity of NK cells against tumor cells. Biochem. Biophys. Res. Commun..

[40] Zhang C., Röder J., Scherer A., Bodden M., Serrahima J.P., Bhatti A., Waldmann A., Müller N., Oberoi P., Wels W.S. (2021). Bispecific antibody-mediated redirection of NKG2D-CAR natural killer cells facilitates dual targeting and enhances antitumor activity. J. Immunother. Cancer.

[41] Silvestre R.N., Eitler J., de Azevedo J.T.C., Tirapelle M.C., Fantacini D.M.C., de Souza L.E.B., Swiech K., Covas D.T., Calado R.T., Montero P.O. (2023). Engineering NK-CAR.19 cells with the IL-15/IL-15Rα complex improved proliferation and anti-tumor effect in vivo. Front. Immunol..

[42] Zhang Y., Zhao Z., Huang L.A., Liu Y., Yao J., Sun C., Li Y., Zhang Z., Ye Y., Yuan F. (2023). Molecular mechanisms of snoRNA-IL-15 crosstalk in adipocyte lipolysis and NK cell rejuvenation. Cell Metab..

[43] Ma S., Caligiuri M.A., Yu J. (2022). Harnessing IL-15 signaling to potentiate NK cell-mediated cancer immunotherapy. Trends Immunol..

[44] Egli L., Kaulfuss M., Mietz J., Picozzi A., Verhoeyen E., Münz C., Chijioke O. (2024). CAR T cells outperform CAR NK cells in CAR-mediated effector functions in head-to-head comparison. Exp. Hematol. Oncol..

