# Supplementary material for: A Novel MICB-Targeting CAR-NK Cells for the Treatment of Pancreatic Cancer
Source: Int J Mol Sci. 2026 Jan 3;27(1):500. doi: 10.3390/ijms27010500 (PMC12786817; doi:10.3390/ijms27010500)
Supplement: Supplementary file 1 [file ijms-27-00500-s001.zip › ijms-3847313-supplementary.pdf]

Supplementary Materials

1. Anti MICB α 3 monoclonal screening and activity identification

1.1 Screening of Anti MICB α 3 monoclonal antibodies

1.1.1 Mouse immunization and hybridoma screening

MICB knockout C57 mice were immunized with pCDNA3.4 plasmid containing the MICB α 3 gene of hMICB, each mouse was immunized with 60μg of the plasmid by intramuscular injection, and a total of 10 mice were immunized. The immunization interval was 2 weeks; 7 days after the three plasmid immunizations, blood was collected, serum was diluted 100-fold, the immune response of mice was detected by using hMICB-overexpressing 293T cells, and mice with obvious immune response were selected to be immunized by tail vein using hMICB-overexpressing 293T cells with an inoculation volume of 1E+07 cells for each mouse. After 3–4 days, the spleens of mice were taken and grinded with 70um sieve mesh and fused with SP2/0 cells by PEG and the hybridoma screening was performed using hMICB-expressing CHO cells. The results are shown in Figure S1. Positive well 03101-6B5 was selected for subcloning, with subclone number 03101-6B5-1A6-1C11.

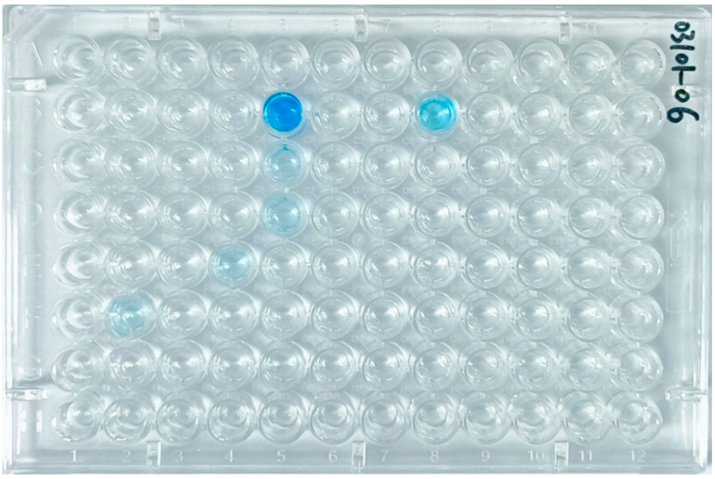

Figure S1. 03101-06-10 post-fusion screening, the most significant response was in well B5.

1.1.2 Preparation of hMICB-CHO high-expressing cell lines:

Collect hMICB high-expressing cell lines, each with approximately 5E+06 cells, with cell viability of 95% or more. 3min(500g centrifugation was performed to collect the cells using an equal volume of pre-cooled PBS containing 1% BSA and washed and centrifuged three times; then, the cells were resuspended at a density of 1E+07cells/mL using pre-cooled PBS containing 1% BSA. PBS was used to resuspend the cells.

1.1.3 03101-6B5-1A6-1C11 Anti-MICB monoclonal hybridoma supernatant-specific reaction.

The cells were numbered as hMICB-CHO-NC and hMICB-CHO-03101-6B5-1A6-1C11 and 100 μL of pre-cooled PBS containing 1% BSA was added to the samples numbered as NC and mixed thoroughly; 100μL of corresponding 03101-6B5-1A6-1C11 monoclonal hybridoma cell line supernatants containing 1% BSA was added to the corresponding flow tubes of 03101-6B5-1A6-1C11, respectively. 6B5-1A6-1C11 monoclonal hybridoma cell line supernatant was mixed thoroughly, all samples were static reaction at 4℃ for 30 min, and then the cells were collected by centrifugation at 500g for 3 min, washed and centrifuged for three times using an

equal volume of pre-cooled PBS containing 1% BSA, and then collected for spare use.

PE-labeled GAM-IgG-PE marker (ab97024) was diluted with pre-cooled PBS containing 1% BSA at a ratio of 1:500 and a total of 2 mL was mixed thoroughly and stored at 4°C for spare use. The hMICB-CHO-03101-6B5-1A6-1C11-treated cells were resuspended by taking the diluted murine secondary antibody dilution according to the amount of 200 μL per tube; hMICB-CHO-NC was added to 200 μL of 1% BSA in pre-cooled PBS to resuspend the cells. The reaction was static at 4°C for 30 min and the cells then were collected by centrifugation at 500 g for 3 min using an equal-volume wash of pre-cooled PBS containing 1% BSA for 3 times.

1.4 Flow assay:

The flow voltage was confirmed using negative control samples. hMICB-CHO-03101-6B5-1A6-1C11 samples were detected using established flow templates and the flow results showed that 03101-6B5-1A6-1C11 had a very good specific response; the results are shown in Figure S2.

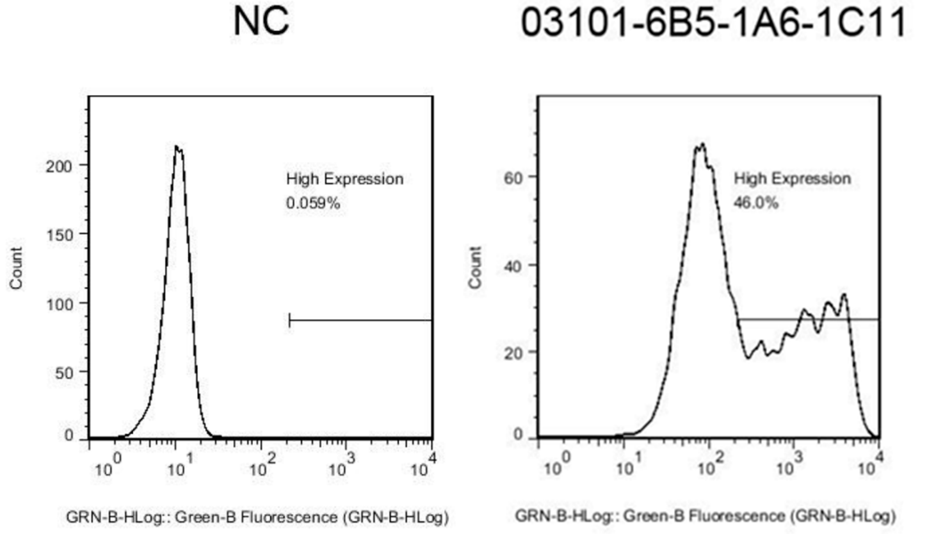

Figure S2.Binding activity of Anti-MICB monoclonal hybridoma supernatant to 293T-hMICB cells

1.2 Anti-MICB 03101-6B5-1A6-1C11 monoclonal antibody affinity test

10 BA LB/c mice were taken and injected with paraffin oil in the peritoneal cavity according to 0.5 mL each for 10 days. Divide the 10 mice into two cages of 5 mice each and inject the pre-treated 03101-6B5-1A6-1C11 monoclonal cell line into the peritoneal cavity according to 1E+06 cells per mouse, start to collect the ascites produced by the mice after 10-12 days, and collect about 10mL of ascites.

The collected ascites was centrifuged at 12,000g for 10 min to collect the supernatant, 50% saturated ammonium sulfate was added, and after thorough mixing, the precipitate was collected by centrifugation at 10,000g for 10 min, resuspended in an equal volume of PBS, and filtered using a 0.45um filter membrane. Using PBS, the protein A affinity chromatography column was equilibrated at a flow rate of 4 mL/min and the pre-treated 03101-6B5-1A6-1C11 was up-sampled and purified at a rate of 4 mL/min, respectively. At the end of the process, it was changed to 0.1 M pH 3.5 acetic acid for elution, the pH of the eluate was adjusted to pH 7.4 using 1 M tris buffer, and the purification was completed by transferring protein A to the protein A. After purification,

the chromatography column was rinsed with 0.1M NaOH buffer; at the end of the process, the column was rinsed with PBS to pH neutral and then rinsed with purified water until the baseline of each assay was stable, and then the column was preserved with 20% ethanol for the protein A chromatography column and the 03101-6B5-1A6-1C11 eluted samples were transferred into 25kD dialysis bags to dialyze them into PBS.
Prepare CHO cells overexpressing human MICB by centrifugation at 300g for 5min, resuspend with PBS, repeat this step twice, and finally adjust the concentration to 3E+06cells/mL with PBS. Ten gradients were set up, and the Anti-MICB monoclonal antibody and the IgG antibody were diluted from 2.5 μg/mL, according to the 2-fold gradient, all the way down to 0.005 μg/mL. The CHO cells overexpressing human MICB were lined up in two rows in each of the transparent 96 round-bottomed wells, 100μL in each well, and then the antibody was added to the cells in order of 1:1 mixing well, for each set of blank wells and negative wells, and then incubated in a refrigerator at 4℃ for 1h; at the end of the incubation, centrifugation was carried out at 500g for 3 min, the step was repeated three times by resuspension with PBS, the PE-labeled goat anti-mouse secondary antibody was diluted to a concentration of 1.500, and 100% of the antibody was added to each well at a concentration of 0.005μg/mL: 500 concentration, 100 μL was added to each well, and the blank wells were not added, put into the 4℃ refrigerator and incubated for 30 min, at the end of the incubation, centrifuged at 500g for 3 min, the step was repeated three times by resuspension in PBS. Finally, 180 μL of PBS was added to each well, the detection was carried out by flow cytometer, and the EC 50 of the Anti-MICB Monoclonal Antibody (03101-6B5-1A6-1C11) was about 0.51 nM. Results are in Figure S3.

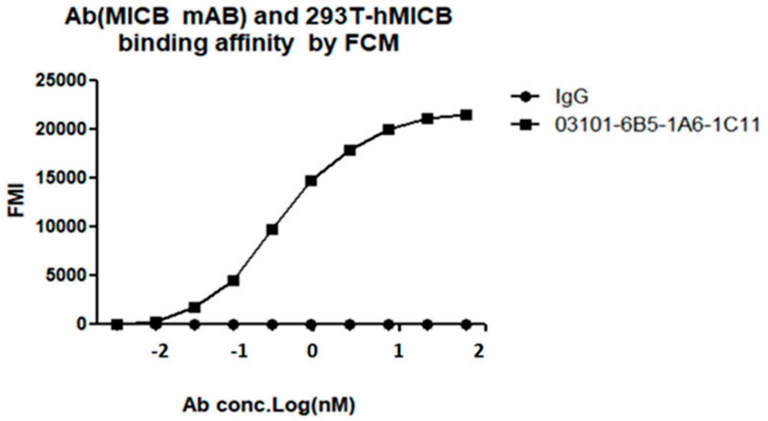

Figure S3. Ab(MICB mAB) and 293T-hMICBbinding affinity by FCM.

1.3 Sequence analysis of 03101-6B5-1A6-1C11 antibody

Clone number	Heavy chain gene	Light chain gene	Subtype
03101-6B5-1A6-1C11	IGHV2-6-7	IGKV4-74	IgG2b

2. Peripheral blood mononuclear cells induced expansion of NK cells in vitro
2.1 Day 0
T25 flasks: Add 5mL of DPBS and 250μL of YC00A to two TC-treated T25 flasks, mix thoroughly, incubate at 37℃ for 2h, discard the supernatant and wash once with 5mL of DPBS, discard the wash solution.
Monocyte inoculation: Add activation medium, 250uL of inducible factor YC00B, 10% Serum

Substitute (1mL) and monocytes to each T25 bottle, total volume 10mL, peripheral blood monocyte cell density 2E6/mL.

2.2 Day 3

Each T25 bottle was supplemented with 19mL of activation medium and 5% Serum Substitute (1mL). At this point the volume in each T25 bottle was 30mL for a total volume of 60mL.

2.3 Day 5

Replenish 140mL of activation medium and 5% Serum Substitute (7mL) and divide the medium and cells from the two T25 bottles equally into two T175 bottles. At this point, each T175 bottle contained 100mL of cell suspension for a total volume of 200mL.

2.4 Day 7

1:1 replenishment of 200mL of amplification medium to a total volume of 400mL with 1% Serum Substitute.

2.5 Day 9

1:1 rehydration, remove 200mL of cell suspension from the original cell culture bag to 2 cell culture bags, and then add 200mL of expansion medium to each of the 2 cell culture bags. At this time, each cell culture bag has 400mL of cell suspension, total volume 800mL.

2.6 Day 11

1:1 replenishment of 800mL of amplification medium.

2.7 Day 13

600mL expansion medium was added.

2.8 Day 16~18

cells were harvested and flow assayed. Results are in Figure S4.

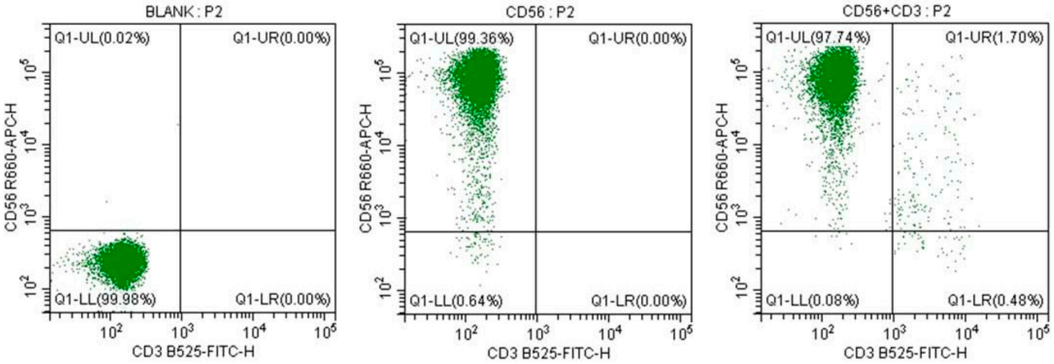

Figure S4. Flow cytometry detection of peripheral blood mononuclear cells for induced expansion of NK cells in vitro.

3. Flow cytometry detection of Anti-MICB-CAR-NK secreted Anti-MICB-scFv

3.1 Inoculate PANC-1 human tumor cells with high expression of MICB in a 6-well plate. The next day, they were digested, collected in 1.5 EP tubes, centrifuged, resuspended in PBS 2 times, with discarded supernatant.

3.2 Set up the background group and Anti-MICB-scFv group. Background group: add 180uLPBS and 20uL of hMICB protein, incubate at room temperature for 1 h. Anti-MICB-scFv group: add 1mL of supernatant collected in 3.1 to 1.5 EP tube, incubate at room temperature for 1 h. Then, centrifuge at 500g for 5 min, discard supernatant, resuspend in PBS twice, discard supernatant. Then 180uLPBS and 20uL of hMICB protein were added and incubated for 1 hour at room

temperature（hMICB Protein-His & hFc Tag, Cat: 10759-H03H）。

3.3 The background and Anti-MICB-scFv groups were incubated with diluted fluorescent secondary antibody for 1 hour at room temperature.（Invitrogen，anti-Human IgG1 Fc Secondary Antibody，Cat: A-10631）。

3.4 Flow cytometry results were as in Figure 1E.

4. **Manuscript data supplement**

4.1 NK cell expansion and culture from hPBMC.

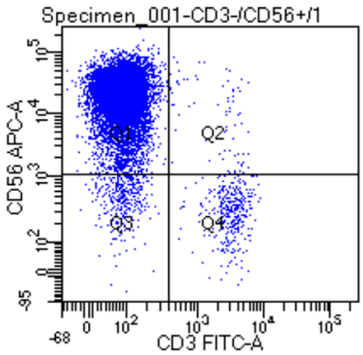

Tube: CD3-/CD56+/1

Population	#Events	%Parent	%Total
All Events	11,051	####	100.0
P1	10,240	92.7	92.7
P2	10,000	97.7	90.5
Q1	9,220	92.2	83.4
Q2	74	0.7	0.7
Q3	350	3.5	3.2
Q4	356	3.6	3.2

Flow cytometry detection of peripheral blood mononuclear cells for induced expansion of NK cells in vitro.

4.2 Efficiency of CAR lentivirus infection in NK cells

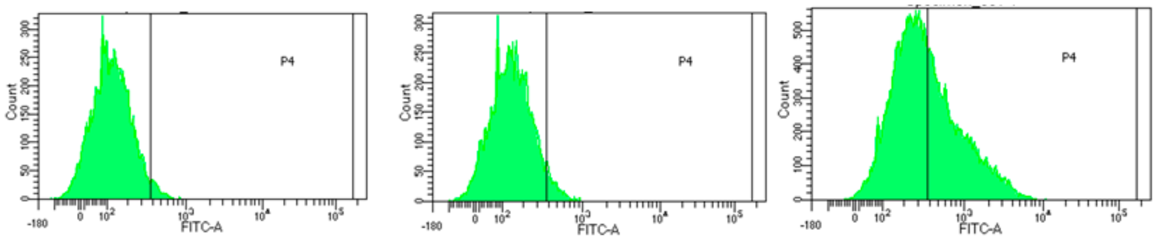

Control NC Anti-MICB-CAR-NK

The efficiency of CAR lentivirus infection of NK is 46%.

4.3 In order to further research if Anti-MICB-CAR-NK promotes tumor cell apoptosis, in addition to PANC-1, we also investigated on the promotion of AsPC-1 tumor cell line by Anti-MICB-CAR-NK (Fig S5).

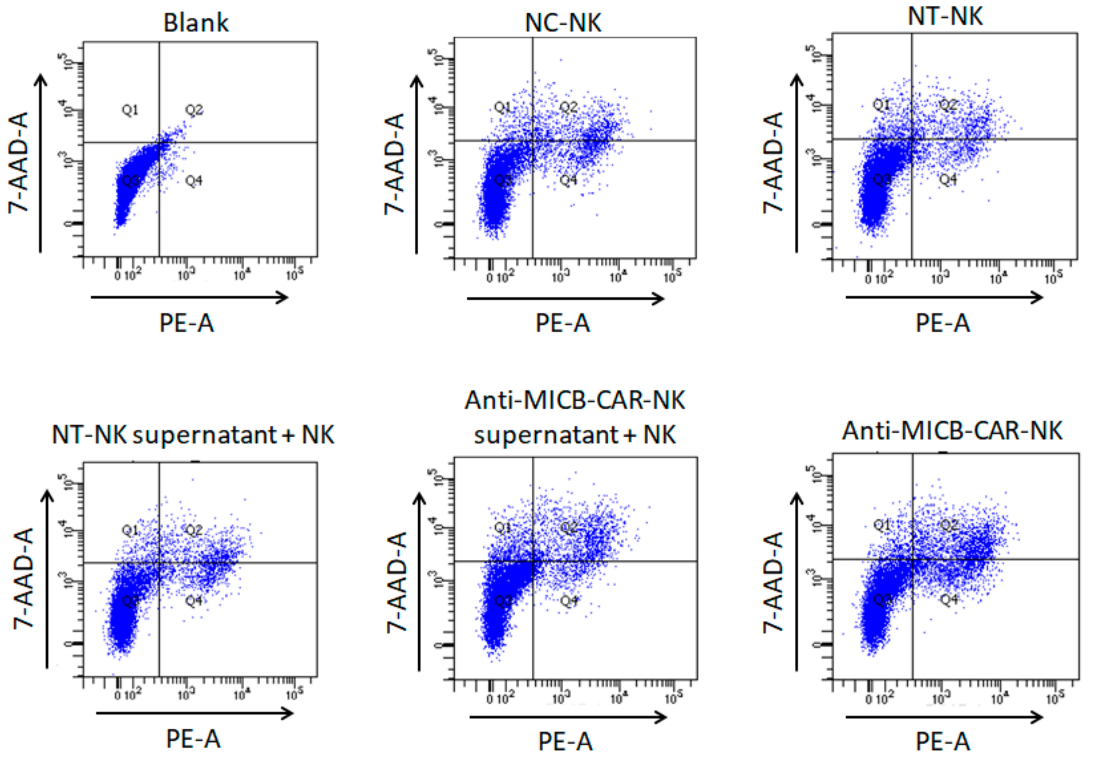

Figure S5. Treated AsPC-1 tumor cells for 24 hours with NC-NK cells, non-transduced NK(NT-NK), NT-NK supernatant + NK cells, Anti-MICB-CAR-NK supernatant + NK cells, and Anti-MICB-CAR-NK cells. The results showed that the viability of AsPC-1 cells was 68.9% after treatment with Anti-MICB-CAR-NK cells and the co-culture of Anti-MICB-CAR-NK supernatant + NK cells that reduced the viability of AsPC-1 cells was 74.7%. In contrast, when treated with NC-NK cells, non-transduced NK (NT-NK), or NT-NK supernatant + NK cells, the survival rate of AsPC-1 cells remained above 80%.
